# Bisphenol A stimulates human prostate cancer cell migration *via* remodelling of calcium signalling

**DOI:** 10.1186/2193-1801-2-54

**Published:** 2013-02-15

**Authors:** Sandra Derouiche, Marine Warnier, Pascal Mariot, Pierre Gosset, Brigitte Mauroy, Jean-Louis Bonnal, Christian Slomianny, Philippe Delcourt, Natalia Prevarskaya, Morad Roudbaraki

**Affiliations:** 1 Inserm, U-1003, Equipe labellisée par la Ligue Nationale contre le cancer, Villeneuve d’Ascq, France; 2 Laboratory of Excellence, Ion Channels Science and Therapeutics; Université Lille I Sciences et Technologies, Villeneuve d’Ascq, France; 3 Laboratoire d’Anatomie et de Cytologie Pathologique du groupement hospitalier de l’Institut Catholique de Lille, Faculté Libre de Médecine, Lille, France; 4 Service d’Urologie de l’hôpital St-Philibert, Lille, France

**Keywords:** Environmental factors, Bisphenol A, Cell migration, Calcium signalling, Ion channels, Orai1, Prostate cancer

## Abstract

Bisphenol A (BPA), the principal constituent of reusable water bottles, metal cans, and plastic food containers, has been shown to be involved in human prostate cancer (PCa) cell proliferation. The aim of the present study was to explore the effect of BPA on PCa cell migration and the pathways involved in these processes. Using the transwell technique, we clearly show for the first time that the pre-treatment of the cells with BPA (1–10 nM) induces human PCa cell migration. Using a calcium imaging technique, we show that BPA pre-treatment induces an amplification of Store-Operated Calcium Entry (SOCE) in LNCaP cells. RT-PCR and Western blot experiments allowed the identification of the ion channel proteins which are up-regulated by BPA pre-treatments. These include the Orai1 protein, which is known as an important SOCE actor in various cell systems, including human PCa cells. Using a siRNA strategy, we observed that BPA-induced amplification of SOCE was Orai1-dependent. Interestingly, the BPA-induced PCa cell migration was suppressed when the calcium entry was impaired by the use of SOCE inhibitors (SKF96365, BTP2), or when the extracellular calcium was chelated. Taken together, the results presented here show that BPA induces PCa cells migration *via* a modulation of the ion channel protein expression involved in calcium entry and in cancer cell migration. The present data provide novel insights into the molecular mechanisms involved in the effects of an environmental factor on cancer cells and suggest both the necessity of preventive measures and the possibility of targeting ion channels in the treatment of PCa cell metastasis.

## Background

Prostate cancer (PCa) is the most common non-cutaneous malignancy diagnosed in men and the metastatic PCa forms represent the second cause of mortality ([Gronberg 2003]; Jemal et al. [2009]). Early PCa requires androgen to survive and to proliferate; this dependence is exploited in the treatment of a disseminated disease, where androgen ablation is the first line of therapeutic intervention. Although these regimens are initially effective, tumors ultimately recur due to reactivation of Androgen Receptor (AR) signalling, causing treatment failure and patient morbidity. Despite the importance of understanding androgen action in the prostate, little is understood about the mechanisms underlying androgen independence, and the means by which the androgen requirement is bypassed in relapsed tumors. As such, identifying the factors affecting the efficacy of androgen deprivation therapy is essential in order to improve the outcome of PCa treatment and thereby to increase patient survival.

Accruing evidence indicates that exposure to environmental compounds, “endocrine disrupting compounds”, or EDCs, may adversely impact human health through multiple mechanisms, including alterations to the hormone receptor function ([Henley and Korach 2006]; Welshons et al. [2003]). In humans, a putative link has been established between an increased abundance of EDCs in the environment and rising hormone-dependent cancer incidence (Huff et al. [1996]). Thus, recent investigations have placed particular emphasis on delineating the consequence of EDC exposure for various tissues including the reproductive tissues.

One such environmental factor is bisphenol A (BPA), a non-planar plasticizer leached in microgram quantities from polycarbonate plastics and epoxy resins into food and water supplies (Welshons et al. [2003]). Studies showed that up to 95% of adults have detectable BPA in their urine (Calafat et al. [2005]), with adult serum concentrations reported to range in nanomolar concentrations [reviewed by (Welshons et al. [2006]). Further, BPA has been shown to be involved in prostate carcinogenesis. A recent animal studies showed that perinatal exposure to BPA at low doses results in increased sensitivity to estrogen as the male animal ages, and to an increased risk of developing PCa (Ho et al. [2006]). At environmentally relevant levels (1nM), BPA has also been identified as a mitogen for a subset of PCa cell lines (Wetherill et al. [2002]; Wetherill et al. [2005]; Wetherill et al. [2006]), in addition to accelerating tumor growth after androgen ablation (Wetherill et al. [2006]). BPA was also shown to induce the growth and resistance to apoptosis of human breast cancer cells, suggesting that hormone-dependent tissues are affected by this environment factor (LaPensee et al. [2010]; Pupo et al. [2012]).

Another aspect of tumor cell evolution is their metastasis, which is the major cause of death from PCa. Invasion of surrounding tissue and vasculature by cancer cells is an initial step in tumor metastasis. Environmental factors such as BPA could impact PCa metastasis by inducing cell migration. However, data on the effects of BPA on human cancer cell migration are lacking.

Accumulating data show that cell proliferation, apoptosis and migration are paralleled by an altered function and/or expression of ion channels involved in the signalling of fundamental cellular mechanisms (Lang et al. [2005]; Prevarskaya et al. [2011]). A ubiquitous Ca^2+^ influx pathway that is activated by intracellular Ca^2+^ store depletion is store-operated Ca^2+^ entry (SOCE), which is activated through a complex interplay between a Ca^2+^ channel at the cell membrane, Orai1, and a Ca^2+^ sensor located in the endoplasmic reticulum, STIM1 ([Courjaret and Machaca 2012]). Recently, a number of known molecular players in cellular Ca^2^ homeostasis, including Orai1, STIM1 and transient receptor potential (TRP) channels have been implicated in tumor cell migration and the metastatic cell phenotype (for review *see* Prevarskaya et al. [2011]). In this context, we previously showed that TRPC1, TRPV6 and Orai1 are the main actors in SOCE in human PCa cells LNCaP (Flourakis et al. [2010]; Vanden Abeele et al. [2003a,, b]).

Here, for the first time, we investigated the impact of BPA on human PCa cell migration and the mechanisms involved in the effects of BPA in these cells. In the latter context, we studied the impact of BPA on calcium signalling, on the expression of ion channels involved in SOCE and human PCa cell migration in the absence of androgens.

## Results

### BPA increases the migration of prostate cancer cells

**Figure 1:**
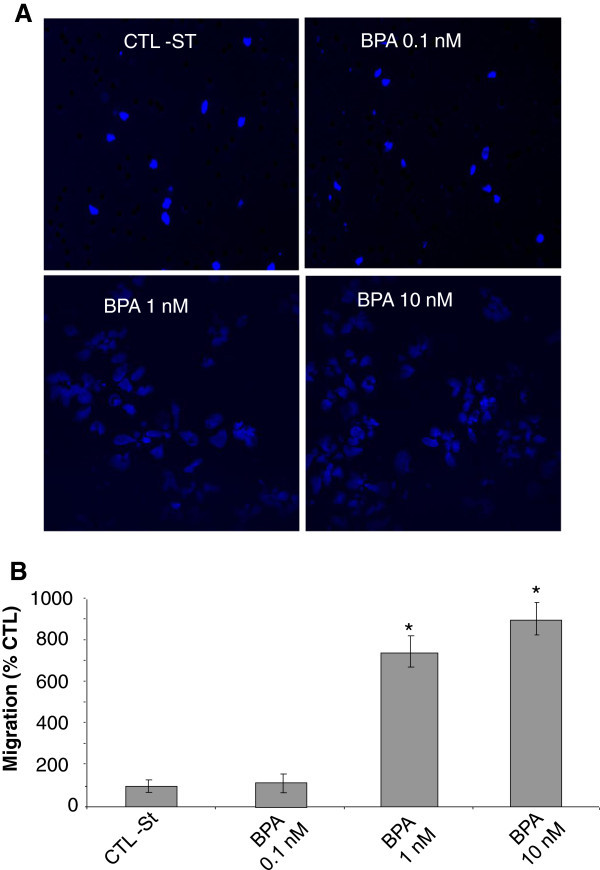
**BPA induces prostate cancer cells migration.** Cell migration was measured *via* the Transwell chamber assay. (**A**) Following the 48 h BPA treatments, LNCaP cells were trypsinized and equal numbers of live cells were plated into inserts and allowed to migrate for 24 h. Cells in the insert (non-migrated cells) were eliminated by scraping and migrated cells were fixed and their nuclei were stained by Hoechst 33342 dye (Blue) and examined under fluorescence microscopy. At least ten different fields per condition were examined and cells were then counted. Experiments were performed in duplicate and repeated at least twice. (**B**) Relative migration (%) ± S.E. (Bar) is shown for the indicated BPA concentrations and the control condition (CTL-ST), where the cells were incubated in CS-RPMI alone. *, *P* < 0.001 relative to CTL condition.

Previous works have clearly shown that BPA induces cell proliferation in androgen-dependent human PCa cells (Wetherill et al. [2005]). In addition to cell growth, cancer metastasis is significantly involved in the progression of the disease and leads to death. Cancer cell migration and invasion play very important roles in cancer metastasis. So, we further studied the effects of BPA on migration and invasion, as well as the related calcium signalling in androgen-dependent and -independent human PCa cells. Migration assay using transwell chambers showed that BPA at low concentrations of 1 and 10 nM significantly increased the migration of LNCaP cells with an increase rate of 800% and 900% respectively, after the cells were treated for 48 h with BPA prior to the migration assays (Figure 1A and 1B). In these experiments, when higher concentrations of BPA (100 nM and 1 μM) were used, the BPA-induced cell migration rate was similar to the one observed with 10 nM (data not shown). We further studied the mechanisms involved in the effects of BPA on PCa cells.

### Effects of BPA on calcium signalling in prostate cancer cells

**Figure 2:**
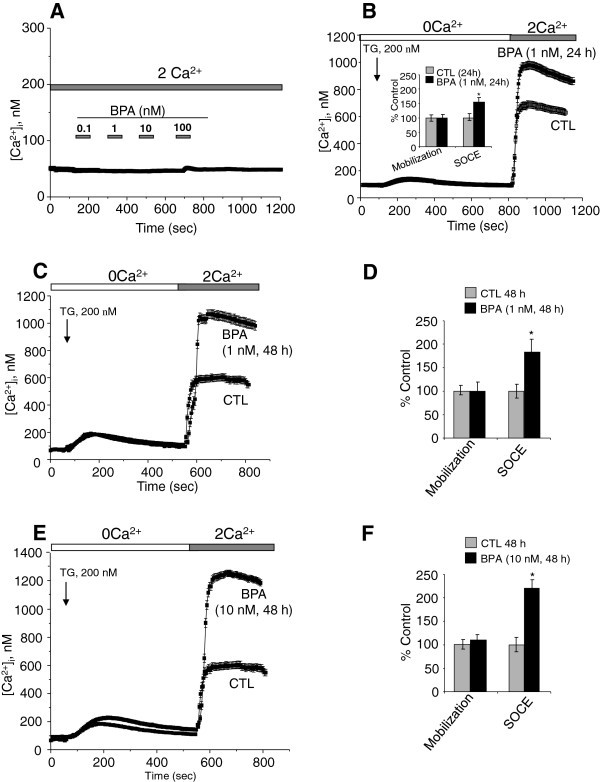
**Effects of BPA on basal calcium and Store-operated Ca**^**2+**^**(SOC) entry (SOCE) in LNCaP cells.** (**A**) Cells were grown on glass coverslips and cultured in complete medium and then the direct effects of BPA at different concentrations on [Ca^2+^]_i_ were studied by calcium imaging. (**B**, **C**, **E**) Cells grown on glass coverslips and cultured in CS-RPMI alone (CTL), or containing BPA at 1 or 10 nM BPA for 24 h (**B**) or 48 h (**C**, **E**) and then processed by calcium imaging to estimate the amplitude of SOCE. For Ca^2+^ recording, cells were treated with 200 nM thapsigargin (TG) in Ca^2+^-free bath solution (0 Ca^2+^) and exposed to 2 mM extracellular Ca^2+^ (2Ca^2+^) as indicated. Each experiment was repeated at least 3 times in duplicate in different cell cultures on a field of 25–40 cells and representative experiments performed on 50–80 cells as mean ± S.E. are presented. A quantification of the SOCE for each experiment is presented in (**D**) and (**F**) (mean ± S.E., n = 50–80 cells), *P < 0.01. The TG application is shown by an arrow and extracellular Ca^2+^ increases from 0 (0 Ca^2+^) to 2 mM (2 Ca^2+^) are marked by horizontal bars.

**Figure 3:**
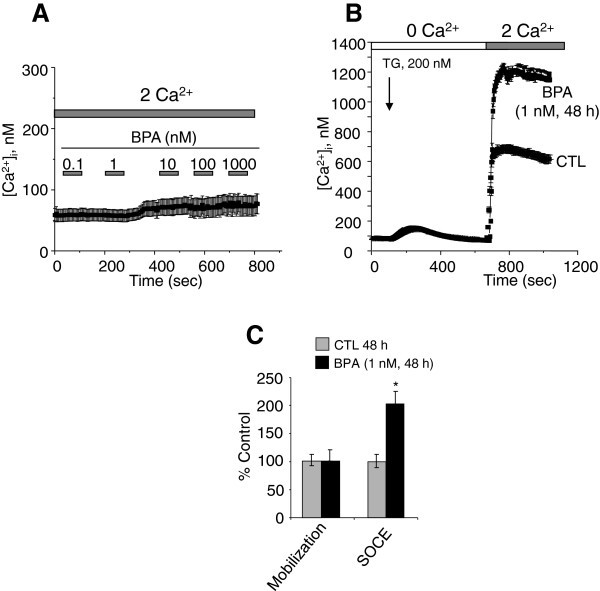
**Effects of BPA on basal calcium and Store-operated Ca**^**2+**^**(SOC) entry (SOCE) in LNCaP C4.2 cells.** (**A**) Cells were grown on glass coverslips and cultured in complete medium and then the direct effects of BPA at different concentrations on [Ca^2+^]_i_ were studied by calcium imaging. (**B**) Cells grown on glass coverslips and cultured in CS-RPMI alone (CTL), or containing BPA at 1 nM BPA for 48h and then processed by calcium imaging to estimate the amplitude of SOCE. For Ca^2+^ recording, cells were treated with 200 nM thapsigargin (TG) in Ca^2+^-free bath solution (0 Ca^2+^) and exposed to 2 mM extracellular Ca^2+^ (2 Ca^2+^) as indicated. Each experiment was repeated at least 3 times in duplicate in different cell cultures on a field of 25–40 cells and representative experiments performed on 50–80 cells as mean ± S.E. are presented. A quantification of the SOCE for experiment (**B**) is presented in (**C**) (mean ± S.E., n = 50–80 cells), *P < 0.01. The TG application is shown by an arrow and extracellular Ca2+ increases from 0 (0 Ca^2+^) to 2 mM (2 Ca^2+^) are marked by horizontal bars. The graphs represent the mean peak amplitudes of calcium mobilization and SOCE ± S.E. of at least three independent experiments and are expressed as the percentage above control value.

Several studies have shown that an increase in intracellular calcium originating from an extracellular source greatly promotes the migration of cancer cells (Saidak et al. [2009]; Yang et al. [2009]). Yang et al. ([2009]) clearly showed that store-operated calcium entry channels (Orai1 and STIM1) are essential for breast tumor cell migration *in vitro* and tumor metastasis in mice. In this context, BPA could induce the activation and/or over-expression of ion channels proteins involved in calcium entry and thus promotes the consequent PCa cell migration. First, we examined the direct effects of BPA on the free cytosolic calcium concentration. When different concentrations of BPA were applied to LNCaP cells, an androgen-dependent PCa cell line (Figure 2A) and to LNCaP-C4.2 cells, which are a more invasive cell line derived from LNCaP cells (Figure 3A), no significant modification of the basal calcium was observed. To determine if BPA can induce a delayed calcium response, LNCaP cells were exposed to a range of the environmental factor concentrations (1nM, 10 nM, 1 μM and 10 μM) and the intracellular calcium concentrations were continuously monitored for 2 to 3 hours. In these experiments, we observed no calcium response to the applications of BPA. These data confirm the absence of a direct effect of BPA on the activity of the ion channels in human prostate cancer cells.

We further examined the modification of the calcium signalling and remodelling of the expression of the ion channels in PCa cells after a pre-treatment with BPA. To verify the possible modification of the calcium signalling in BPA-treated cells, a test, as described in the following section, was used to compare the rate of calcium entry in control and in BPA-treated cells.

### Ca^2+^ entry, but not Ca^2+^ release, is increased in BPA- treated LNCaP cells

We previously showed that the application of the store-depleting SERCA inhibitor thapsigargin (TG) induces a calcium mobilization from intracellular stores and a calcium entry due to SOCE in human PCa cells (Lallet-Daher et al. [2009]).

The two phases of free intracellular calcium concentration ([Ca^2+^]i) changes were separated using a Ca^2+^ add-back protocol. The addition of the store-depleting SERCA inhibitor thapsigargin (TG, 200 nM) in nominally Ca^2+^-free solution was followed by rapid, transient increases in [Ca^2+^]_i_, as measured by calcium imaging (Figure 2B) These increases are due to the mobilization of Ca^2+^ from internal stores. A subsequent addition of Ca^2+^ to the extracellular bath resulted in a rapid and sustained increase in cytosolic Ca^2+^ due to SOCE. Analyses were also performed on both the rate of the TG-induced calcium mobilization and the amplitude of SOCE in CTL cells *versus* those treated with different concentrations of BPA for 24 and 48 h. In these analyses, the amplitude and the decay of the calcium mobilization were not significantly different between CTL and treated cells (Figure 2B, 2D and 2F). Interestingly, in these experiments, we observed that when the cells were cultured in a steroid-free medium (CS-RPMI), the amplitude of the SOCE was significantly lower (40 to 50%) than that developed in cells cultured in normal RPMI containing steroids (data not shown). TG-mediated Ca^2+^ signalling was then studied in LNCaP treated with BPA for 24, and 48 h. At 24 h (Figure 2B), the amplitude of the Ca^2+^ entry (SOCE) was 40 ± 15% higher than that developed in the cells cultured for the same periods in CS-RPMI alone (CTL). These effects of BPA reached 85 ± 19% and 122 ± 19% when the cells were pre-treated for 48 h by BPA 1 nM (Figure 2C and 2D) and 10 nM (Figure 2E and 2F) respectively. Similar SOCE amplifications were observed for LNCaP-C4.2, a more invasive cell line derived from LNCaP cells (Figure 3B and 3C). These observations suggest that BPA modulates the expression of the ion channels involved in the SOCE in human PCa cells. The identification of these ion channels could allow a schematization of the mechanism by which BPA modulates these cancer cells migrations.

### BPA up-regulated ion channels expression in prostate cancer cell lines

Our previous studies showed that of several ion channels, it was mainly the calcium channels (Orai1/STIM1, TRPV6) and potassium channels (IK_Ca1_, BK_Ca_) that were involved in the generation of the SOCE in human PCa cells (Flourakis et al. [2010]; Lallet-Daher et al. [2009]; Vanden Abeele et al. [2003a,, b]). Thus, experiments were designed to explore the impact of a 48 h exposure of the cells to BPA on the expression of these calcium and potassium channels involved in the SOCE. In the present study, in order to eliminate the impact of steroids, and androgens in particular, the experiments were performed in Phenol red-free RPMI 1640 containing charcoal-stripped Foetal Calf Serum (FCS) (CS-RPMI or -ST), a steroid-deprived medium. We have previously shown that the expression of Orai1 was at least decreased by 90% in this steroïds-free medium (Flourakis et al. [2010]).

**Figure 4:**
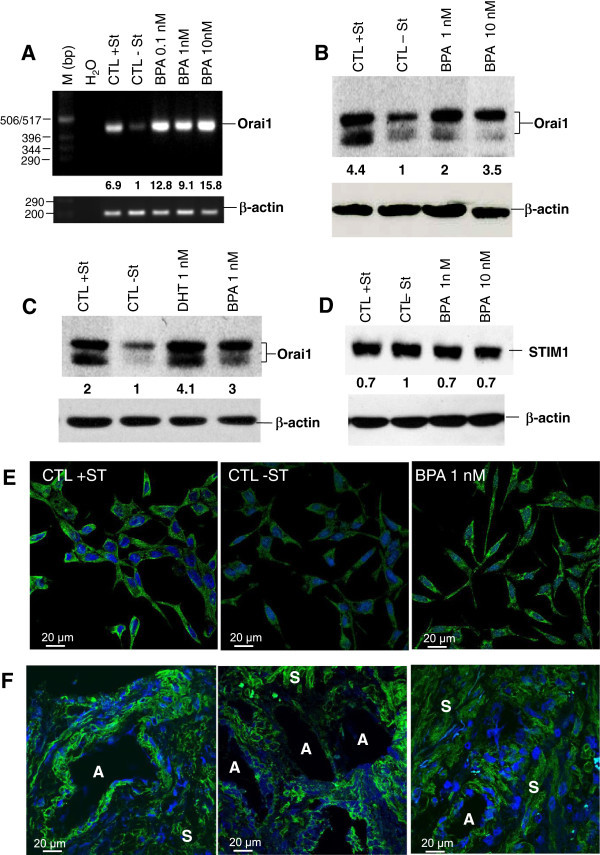
**Effects of BPA on Orai1 and STIM1 expression in prostate cancer cells.** (**A**), Total RNA was extracted and semi-quantitative RT-PCR experiments performed to study the expression of Orai1 (406 bp) mRNA using specific primers. β-actin mRNA expression (212 bp) was used as an internal standard. PCRs were carried out as described in *Methods* using 40 ng ARN equivalent cDNA from CTL and treated samples. H2O and samples without reverse transcriptase (not shown) were used as negative controls. Amplified fragments were resolved on 1.5% agarose gel by electrophoresis and visualized by EtBr staining.M, 1-kb DNA ladder, a molecular weight marker. (**B**, **C**, **D**), Effects of BPA on the ion channels' protein expression in LNCaP cells. Following the 48 h BPA treatments, total proteins were extracted and 10 μg proteins were analysed on 10% acrylamide gel (SDS-PAGE), transferred to PVDF membranes. Immuno-blots were then performed as described in *Methods* for the detection of Orai1 and STIM1. β-actin expression was used for the loading control of the samples. Experiments were performed at least twice in two independent cell cultures and representative figures are presented. The fold variation of the Orai1 and STIM1 proteins in different samples normalized to β-actin expression is shown under the panel. (**E**), Immunofluorescence studies of the effects of BPA on the expression of Orai1 in LNCaP. Cells were cultured for 48 h in the complete medium containing steroids (CTL + ST) or in the same medium without steroids (CS-RPMI, CTL -ST) or in CS-RPMI containing 1 nM BPA. (**F**), Expression of Orai1 protein in human PCa tissue. PCa tissue Cryosections were processed by Immunofluorescence for the detection of Orai1. *S*, Stroma; *A*, Acinus.

As shown in Figure 4, the rate of Orai1 transcript (Figure 4A) and protein (Figure 4B) was significantly lower when the cells were incubated in CS-RPMI, suggesting that Orai1 expression is steroid-dependent. Interestingly, when the cells were treated with 0.1 to 10 nM BPA, the Orai1 protein level was much higher at the mRNA (Figure 4A) and protein (Figure 4B) levels. Orai1 protein appears as a doublet band representing different glycosylated forms of the protein, as previously described by Gwack et al. ([2007]). We have previously shown that the expression of Orai1 was modulated by the AR pathways (Flourakis et al. [2010]). In the present work, the AR agonist (DHT) was used as a positive CTL for the induction of Orai1 protein expression, as observed by western-blotting. As shown in Figure 4C, the expression of Orai1 induced by the activation of the Androgen Receptor by 1 nM DHT was mimiked by BPA. This would suggest that the effects of BPA on Orai1 expression could be at least partly mediated by activation of the AR. In these experiments, BPA failed to significantly affect the expression of the Orai1 partner, STIM1 (Figure 4D). The up-regulation of Orai1 protein by BPA is confirmed by immunofluorescence studies performed on LNCaP cells. The incubation of the cells in CS-RPMI (−ST) reduced Orai1 staining, whereas the 1 nM BPA treatments of cells for 48 h induced intense Orai1 protein staining (Figure 4E). As these experiments on Orai1 proteins were performed on the LNCaP PCa cell line, we studied the expression of Orai1 protein in 3 grade 3 human PCa tissues using immunofluorescence. As shown in Figure 4F, Orai1 immunostaining was observed in epithelial cells of the acini, as well as in stromal cells, suggesting a role for the protein in both the epithelial and stromal cells compartments of PCa tissues.

**Figure 5:**
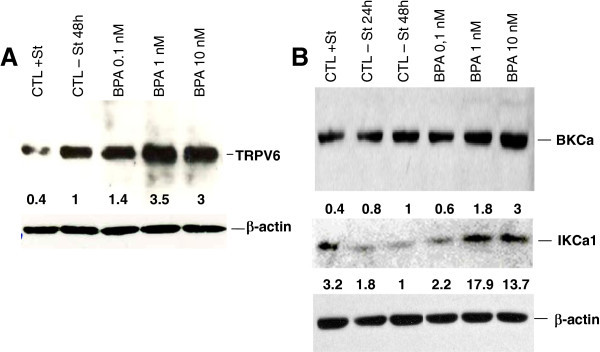
**Effects of BPA on the ion channels proteins expression in LNCaP cells.** Following the 48 h BPA treatments, total proteins were extracted and 10 μg proteins were analysed on 10% acrylamide gel (SDS-PAGE), then transferred to PVDF membranes. Immuno-blots were then performed as described in *Methods* for the detection of TRPV6 (**A**), BK_Ca_ and IK_Ca1_ (**B**). β-actin expression was used for the loading control of the samples. Experiments were performed at least twice in two independent cell cultures and representative figures are presented. The fold variation of the proteins in different samples normalized to β-actin expression is shown under the panel.

Further experiments dealing with the modulation of other calcium and potassium channels potentially involved in SOCE in LNCaP cells showed a clear modification of their expression in BPA-treated cells. In the same manner, the expression of the TRPV6 calcium channel (Figure 5A) and those of BK_Ca_ and IK_Ca1_ Ca^2+^-activated potassium channels (Figure 5B) were clearly induced. The up-regulated potassium channels (BK_Ca_ and IK_Ca1_), by hyperpolarizing the membrane potential, may be implicated in the BPA-induced amplification of SOCE, by promoting significant calcium entry through Orai1 and TRPV6.

### Involvement of Orai1 in BPA-induced modification of calcium signalling

**Figure 6:**
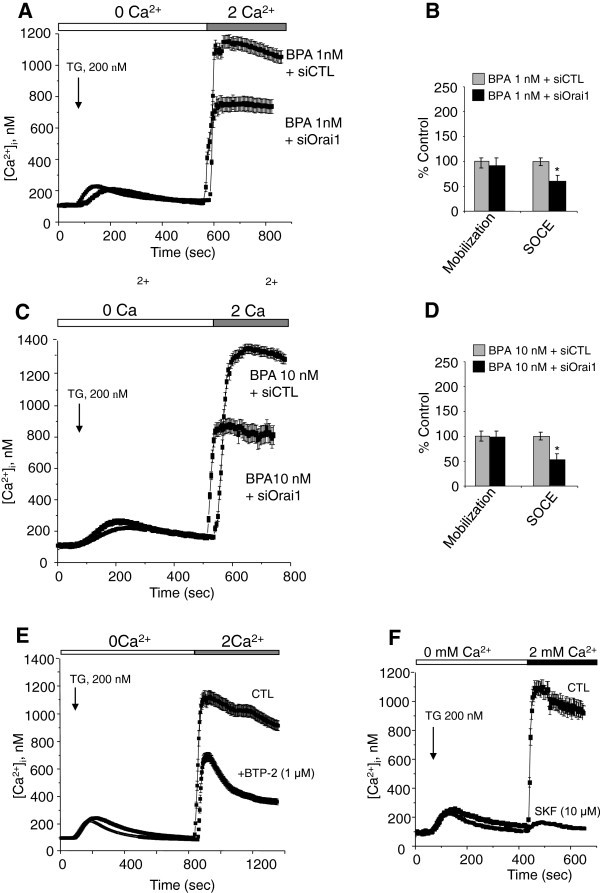
**Effect of Orai1 knockdown on Ca**^**2+**^**entry induced by BPA treatments in LNCaP cells (A, B, C, D).** Forty-eight hours prior to recording Ca^2+^ signals by calcium imaging, LNCaP cells were divided into paired groups and transfected with control siRNA (siCTL), or siOrai1 in the presence of 1 or 10 nM BPA. For Ca^2+^ recording, cells were treated with 200 nM thapsigargin (TG) in Ca^2+^-free bath solution (0 Ca^2+^) and exposed to 2 mM extracellular Ca^2+^ (2 Ca^2+^) as indicated. Using the same protocol described above, pharmacological tools were used to study the involvement of Orai1 in BPA-induced SOCE amplification. LNCaP cells were treated with BPA (1nM, 48h) and then the TG-induced SOCE was studied in the presence or absence of BTP2 (2 μM) (**E**), an inhibitor of Orai1 or SKF96365 (10 μM) (**F**), a broad spectrum inhibitor of ion channels involved in SOCE. Each experiment was repeated at least 3 times in duplicate in different cell cultures on a field of 25–40 cells and representative experiments performed on 50–80 cells as mean ± S.E. are presented. A quantification of the SOCE for each experiment is presented in (**B**) and (**D**) (mean ± S.E., n = 50–80 cells), *P < 0.001. The TG application is shown by an arrow and extracellular Ca^2+^ increases from 0 (0 Ca^2+^) to 2 mM (2 Ca^2+^) are marked by horizontal bars.

It has previously been shown that Ca^2+^ influx is essential for the migration of various cell types, including tumor cells ([Komuro and Rakic 1993]; [Marks and Maxfield 1990]; Nishiyama et al. [2003]; [Yang and Huang 2005]). In addition, recent data has clearly shown that Orai1 and STIM1, both of which involved in store-operated calcium entry, are essential for tumor cell migration *in vitro* and tumor metastasis in mice (Yang et al. [2009]). Thus, the BPA-induced up-regulation of Orai1 expression might be involved in the effects of the environmental factor on cell migration. In this context, we studied the involvement of the Orai1 protein in the effects of BPA on calcium signalling in LNCaP cells. The cells were treated with Orai1 targeting siRNA (siOrai1, 20 nM) for 48 h during the BPA treatments of the cells. Then, calcium imaging experiments were performed using the protocol described in Figure 2. As shown (Figure 6A-D), the treatment of the cells by siOrai1 suppressed the BPA-induced SOCE amplification by 60 to 80%. As described above, in the present study, in order to eliminate the impact of steroids, and androgens in particular, the experiments were performed in CS-RPMI (−ST), a steroid-deprived medium. In this medium, the expression of Orai1 was at least decreased by 90% (Flourakis et al. [2010]). As expected, the siOrai1 treatments of the cells without BPA treatments did not modify the amplitude of the TG-induced calcium entry. In these experiments, the siTRPV6 treatments in the presence of BPA 1 and 10 nM failed to inhibit the BPA-induced amplification of the calcium entry induced by TG (data not shown).

Pharmacological tools were also used to study the involvement of Orai1 in BPA-induced SOCE amplification. LNCaP cells were treated with BPA (1nM, 48h) and then the TG-induced SOCE was studied in the presence or absence of the inhibitors. When the experiments were performed using a pyrazole derivative, BTP2 (2 μM), known to inhibit calcium channels involved in SOCE including Orai1 (Eltit et al. [2010]), the TG-induced SOCE amplitude in BPA-treated cells was inhibited by at least 40% (Figure 6E). In the same manner, when the experiments were performed in the presence of an inhibitor of store-operated Ca^2+^ entry (SKF96365, 10 μM), the TG-induced SOCE was almost completely inhibited in BPA-treated cells (Figure 6F). Taken together, these data suggest that the up-regulation of Orai1 is involved in the amplification of the calcium entry induced by BPA.

### Involvement of calcium entry in BPA-induced cell migration

**Figure 7:**
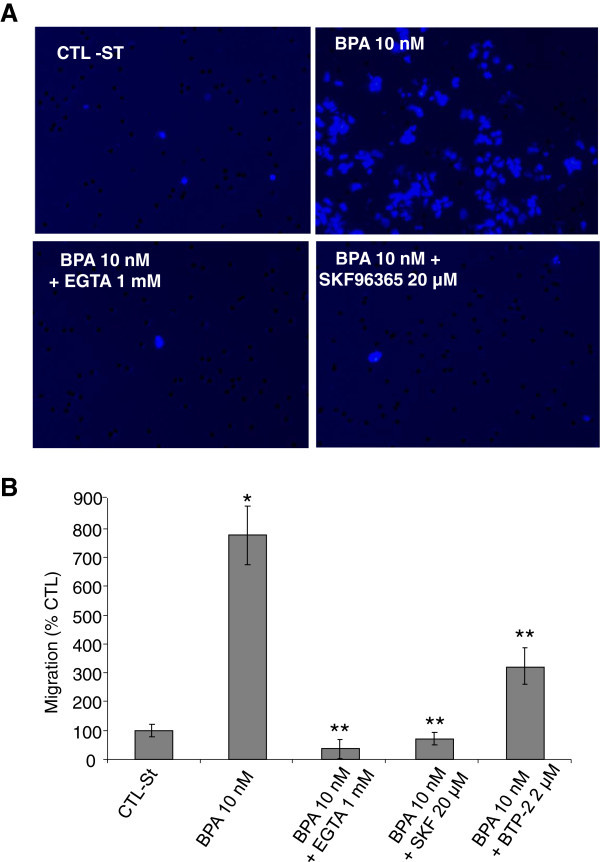
**BPA-induced SOCE is involved in prostate cancer cells migration.** Cell migration was measured via the Transwell chamber assay. (**A**) Following the 48 h 10 nM BPA treatment for, LNCaP cells were trypsinized and equal numbers of live cells were plated into inserts and allowed to migrate for 24h in the presence or absence of BPA 10 nM, with or without the presence of a Ca^2+^ chelator EGTA (1 mM) or the SOCE inhibitors (SKF, 20 μM; BTP-2, 2 μM). Cells in the insert (non-migrated cells) were then eliminated by scraping and migrated cells were fixed and their nuclei were stained by Hoechst 33342 dye (Blue) and examined under fluorescence microscopy. At least ten different fields per condition were examined and the cells counted. Experiments were performed in duplicate and repeated at least twice. (**B**) Relative migration (%) ± SE (Bar) is shown for the control condition (CTL-ST), where the cells were incubated in CS-RPMI alone, and for BPA (10 nM) in the presence or absence of the EGTA or SOCE inhibitors. *, *P* < 0.001 relative to CTL condition; ** *P* < 0.001 BPA 10 nM *versus* BPA 10 nM + inhibitors.

We showed that Orai1 protein was up-regulated due to BPA treatments of the LNCaP cells and that Orai1 was involved in the amplification of the calcium entry into these cells. To further investigate the correlation between Ca^2+^ influx, SOCE and cell migration induced by BPA, we studied the effect of blocking Ca^2+^ influx on LNCaP cell migration. Using a transwell migration assay, we observed that when the cells were incubated in a medium depleted in calcium by the addition of 1 mM EGTA (Calcium chelating agent), thereby reducing the extracellular free calcium to nominally 200 nM, the BPA (10 nM)-induced migration of the LNCaP cells was completely inhibited (Figure 7A and B). In the same manner, in order to study the involvement of SOCE in BPA-induced migration, cells were treated with an inhibitor of store-operated Ca^2+^ entry (SKF96365, 20 μM) in the presence of BPA (10 nM). As shown in Figures 7A and 7B, SKF96365 blocked all BPA-induced cell migration. When the experiments were performed using BTP2 (2 μM), known to inhibit the calcium channel Orai1 involved in SOCE (Figure 7B), the BPA-induced cell migration was inhibited by 63 ± 11%. These pharmacological inhibitor data show that blocking Ca^2+^ influx inhibits the BPA-induced migration of LNCaP cells, and that the store-operated Ca^2+^ entry channels including Orai1 are indeed involved in the effects of BPA on cell migration.

### BPA increases the migration of androgen-independent prostate cancer cells

**Figure 8:**
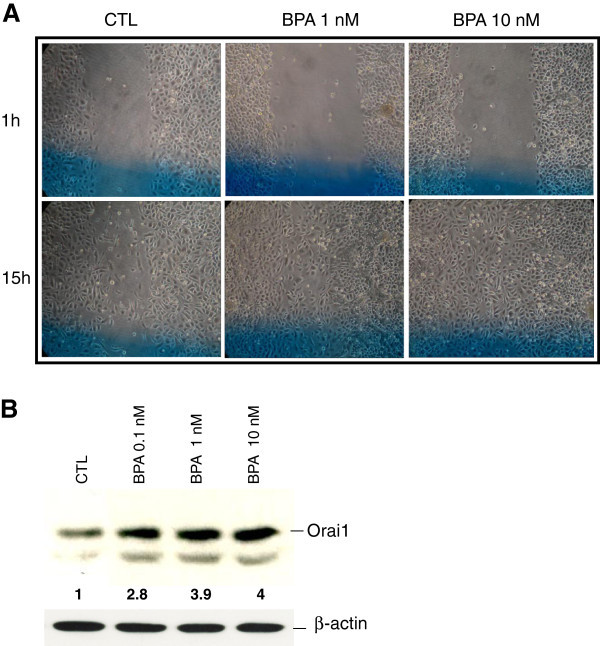
**BPA induces the cell migration of androgen-independent prostate cancer cells.** (**A**) Cell migration was studied via the *in vitro* wound-healing assay. The PC-3 cells grown to confluent monolayers were incubated for 48 h in CS-RPMI, complemented or not by BPA at different concentrations. The cells were then wounded using a sterile tip and incubated in the corresponding media for an additional 15 h before the microscopic examinations of the wound-healing of the cells. Wound-healing was examined 1 and 15 hours after scratching, in at least 3 different previously identified (Blue line) fields per scratch and representative figures for each condition are presented here. (**B**) Western blot analysis of whole cell lysate proteins of Orai1, in PC-3 cells treated with BPA at different concentrations. The figure is a representative experiment showing the variation of Orai1 protein under BPA treatments. β-actin was used as a sample loading control. The fold variation of the Orai1 protein in different samples normalized to β-actin expression is shown under the panel. Results shown are representative of at least three independent experiments.

Previous studies reported by Wetherill et al. ([2005]) showed that low concentrations of BPA induced proliferation in AR-dependent cells, but that the environmental factor failed to affect the growth of AR-negative, androgen-independent PCa cell lines (PC-3 and DU-145). In the present study, we examined the effects of BPA on the migration of the androgen-independent PCa cell line PC-3. The cells were incubated for 48 h in CS-RPMI, both complemented or not by BPA at different concentrations. The cells were then wounded using a sterile tip and incubated in the corresponding media for an additional 15 h before microscopic examinations of the wound healing of the cells. As shown in Figure 8A, these *in vitro* scratch tests of PC-3 cells showed an increase in cell wound closure in response to BPA, suggesting the induction of cell migration by BPA in the androgen-independent PCa cell line PC-3. In order to study the effects of BPA on Orai1 protein expression, Western blot experiments were performed on total proteins of the PC-3 cells treated for 48 h with BPA at varying concentrations. As shown in Figure 8B, BPA induced a dose-dependent expression of Orai1 in PC-3 cells.

## Discussion

It has been suggested that the environmental factor BPA may play an important role in the initiation (Ho et al. [2006]) and progression of PCa and in hormonal therapy bypass (Wetherill et al. [2005]). At the level of PCa cells, BPA was able to induce androgen-independent tumor cell proliferation and reduced therapeutic efficacy in xenograft models (Wetherill et al. [2006]). While these data point toward the potential for BPA to assist tumor cells in escaping therapy, the molecular mechanisms of this process were not well-known.

This report demonstrates for the first time that the environmentally relevant concentrations of BPA (1–10 nM) induce cell migration by modulating the cell calcium signalling. These data highlight the previously unrecognized action of BPA in the progression of human PCa, thereby providing strong support for the growing recognition of the adverse effects of BPA on human health.

Invasion and metastasis are major events underlying cancer morbidity and mortality (Molloy and Van ’t [Veer 2008]). Because of the widespread metastasis in advanced cancer patients, where a resistance is observed to conventional therapies, the mortality rate remains extremely high and warrants new strategies to intervene in the metastatic cascade. Thus, an enhanced understanding of the molecular events in the pathogenesis of PCa will offer improved diagnosis, prognosis, therapy and prevention measures of the disease, that will ultimately help us to eliminate PCa metastasis. A common regulatory point in several signal transduction pathways is intracellular calcium homeostasis. One approach could be to focus on the intracellular signalling pathways underlying the metastatic process. Several data have clearly shown the involvement of calcium entry in cancer and non-cancer cell migration (Bisaillon et al. [2010]; Li et al. [2011]; Schaff et al. [2010]; Yang et al. [2009]).

In the present work, we present conclusive evidence for the first time that the pre-treatment of human PCa cells with environmentally relevant concentrations of BPA (1–10 nM) induces their migration (Figure 1). By calcium imaging technique, we show that BPA pre-treatment induces an amplification of Store-Operated Calcium Entry (SOCE) in LNCaP cells (Figure 2). RT-PCR and Western blot experiments allowed us to identify those ion channel proteins up-regulated by BPA pre-treatments. These channels include Orai1, a protein known to constitute an important actor in SOCE in various cell systems including human PCa cells (Figure 4). Meanwhile, in our studies, we failed to observe any direct effect of BPA on the rate of basal calcium (Figure 2) whereas in other cell systems, BPA or its derivatives induced a calcium increase. In TM4 Sertoli cells, a direct application of a derivative compound of BPA, Tetrabromobisphenol A (TBBPA), a commonly used brominated flame retardant (BFR), induced an increase in the basal free calcium rate originating from internal stores (Ogunbayo et al. [2008]). In pituitary tumor cells (GH**3**/B**6**/F10 rat Somatomammotropes), Kochukov et al. ([2009]) showed that BPA at a 1 nM concentration induced a great increase in Ca^2+^ oscillation frequency, the activation of MAPK pathways (ERK1/2) and subsequently a PRL release (Kochukov et al. [2009]). Similar results were reported by Bulayeva et al. ([2005]) and Wozniak et al. ([2005]) where the authors demonstrated that the BPA-induced Ca^2+^ influx was strictly dependent on membrane Estrogen Receptor (mER-α) and mediated by L-type voltage-gated Ca^2+^ channels in pancreatic β cells (Bulayeva et al. [2005]; Wozniak et al. [2005]). The LNCaP cells used in our present work do not express L-type voltage-gated Ca^2+^channels (non-excitable cells). This is probably the reason why a direct application of BPA on LNCaP and LNCaP C4.2 cells failed to induce a direct calcium response.

In the present work, when the human PCa cells were pre-treated with BPA (1–10 nM), an increase in SOCE and a remodelling of ion channel expression was observed. The alterations of the ion channel expression could be mediated by a stimulation of a signal transduction pathways leading to the activation of nuclear transcription factors. Published data suggest several transduction pathways activated by BPA. In PCa cells, BPA has been shown to be an agonist for mutant androgen receptor (AR-T877A) expressed in recurrent PCa (Wetherill et al. [2002,, 2005,, 2006]), and in the LNCaP cell line used in our studies. According to the authors, BPA induces cell proliferation in cells expressing the mutated AR. The clinical ramifications of BPA activating tumor-derived mutant ARs and inducing androgen-independent tumor cell proliferation may be substantial, as BPA can reduce therapeutic efficacy in xenograft models (Wetherill et al. [2006]). In our experiments, the DHT induced the expression of Orai1 (Figure 4C) and BPA appears to mimic these effects on the canonical AR ligand (DHT). However, all the effects of BPA could not be mediated by the activation of AR. In a recent work, Hess-Wilson et al. ([2007]) showed clearly that BPA and DHT elicited distinct transcriptional signatures in PCa cells expressing the BPA-responsive mutant AR-T877A, even if some common genes were activated by both DHT and BPA in LNCaP cells (Hess-Wilson et al. [2007]). These observations could explain the cell migration and Orai1 expression induced by BPA in androgen-independent human PCa cells PC-3 (Figure 8), where the AR is absent. BPA could thus activate other signal transduction pathways than the AR activation, to induce the effects observed in our studies on androgen-insensitive PCa cells. The study of the involvement of other transduction pathways than those involving the AR receptor (growth factor signalling pathways, …) in the effects of BPA in androgen-dependent and androgen-independent cells needs further extensive investigations in the future. In this context, BPA was reported to induce the phosphorylation of extracellular signal–regulated kinase (ERK), *c-Jun* N-terminal kinase (JNK), and nuclear translocation of the nuclear factor (NF)-*κ*B, in mouse hippocampal HT-22 cells (Lee et al. [2008]). Interestingly, functional NF-*κ*B-binding sites in promoter regions of STIM1 and Orai1 have been identified and the expression of the Orai1 calcium channel was reported to be positively modulated by NF-κB (Eylenstein et al. [2012]). Subsequently, the store-operated Ca^2+^ entry was similarly increased by overexpression of p65/p50 or p65/p52, and decreased by treatment with the NF-*κ*B inhibitor, Wogonin. BPA could thus interfere with the growth factors' signal transduction to activate the PI3K/AkT pathway and thereby induce the activation of the NF-*k*B transcription factor, leading in turn to an up-regulation of the expression of ion channels including Orai1. In this context, authors have shown that the activation of mER-α induces the activation of the PI3K/Akt signalling cascade (Simoncini et al. [2003]; Stirone et al. [2005]) and BPA is shown to activate mER-α (Bouskine et al. [2009]; Quesada et al. [2002]). The activation of the mER-α in androgen-dependent (LNCaP) and androgen-independent (PC-3) PCa cells may thus induce the PI3K/Akt signalling cascade which leads to the activation of the NF-kB transcription factor and Orai1 gene expression. Several works have also demonstrated the stimulation of the PKA/CREB pathways by nanomolar concentrations of BPA through the activation of mER-α (Bouskine et al. [2009]; Quesada et al. [2002]). However, the involvement of this pathway in the expression of ion channels needs further investigation. As shown in Figure 5, BPA pre-treatment induced an increase in BK_Ca_ and IK_Ca1_ Ca^2+^-activated potassium channel expression in LNCaP cells. We previously showed that the IK_Ca1_ Ca^2+^-activated potassium channels are involved in SOCE in LNCaP and PC-3 human PCa cells (Lallet-Daher et al. [2009]). These potassium channels could constitute a functional complex with Orai1 protein to promote calcium entry and cell migration. A study is in progress in our lab to show the functional up-regulation and involvement of these Ca^2+^-activated potassium channels (IK_Ca1_ and BK_Ca_) in cell migration in the BPA-treated LNCaP and PC-3 cancer cells.

Receptor-mediated activation of phospholipases C by the factors present in the serum leads to IP_3_-mediated depletion of Ca^2+^ from the ER, which in turn stimulates Ca^2+^ influx through the plasma membrane involving Orai1/STIM1 complex formation (Varnai et al. [2009]). Recent works have elegantly demonstrated that STIM1, Orai1, and SOCE play critical roles in the migration of a number of cell types in cancer and non-cancer cells (Bisaillon et al. [2010]; Li et al. [2011]). These observations support our data where BPA, by up-regulating the ion channels expression increases the SOCE developed by PCa cells in response to factors present in serum.

Our data show clearly that the BPA-induced cell migration is dependent on the calcium entry and the use of pharmacological tools suggests the involvement of SOCE channels in the effects of BPA on cell migration (Figure 7). Increase in cytoplasmic calcium induced by BPA may have several types of impacts which trigger cell migration, including the induction of the up-regulation of their gene and protein expression, secretion and the activation of the enzymes such as metalloproteinases (MMP2, MMP9), which are involved in cell migration. The MMP proteins are clearly shown to be dependent on calcium for their expression (calcium/Calcineurin/NFAT, …), their processing and their activity (Collier et al. [1988]; Mukhopadhyay et al. [2004]; Stetler-Stevenson et al. [1989]). These observations suggest that the increase in calcium entry induced by BPA pre-treatment could promote all these processes leading to cell migration.

For the first time, we also demonstrate the expression of Orai1 proteins in human PCa tissues (Figure 4F). As shown, a strong immuno-staining of Orai1 protein was found in epithelial cells of the acini and also in the stromal cells. Thus, stromal cells could also be influenced by BPA impregnation. Given the importance of the epithelium-stroma (reactive stroma) in the progression of cancer, the potential effects of BPA on calcium signalling and on the secretion of growth factors by these cells need further investigation.

## Conclusions

BPA is consistently detected in almost all individuals in developed nations (Welshons et al.[2006]), suggesting that humans are continuously exposed to BPA. In addition, the rapid metabolic clearance of BPA and its detectable levels in human blood and urine suggest that the intake of BPA may be higher than indicated by diverse studies and that long-term daily intake may lead to its bioaccumulation, leading to adverse effects on human health and on cancer progression.

These observations suggest that the BPA concentrations used in the present study are attainable in humans. The present data provide novel insights into the way in which the molecular mechanisms involved in the effects of environmental factors can promote the progression of the cancer in an androgen-independent manner. Our work also highlights the urgency of taking preventive measures and suggests some potential therapeutic opportunities of targeting the ion channels involved in SOCE (Orai1) in order to prevent the PCa cell growth and metastasis.

## Methods

### Chemicals and antibodies

Bisphenol A (BPA) was obtained from Sigma-Aldrich and dissolved in DMSO. Antibodies raised against human ion channel proteins were obtained from commercial sources as follows: Rabbit anti-Orai1 (ProSci Inc.), Rabbit anti-STIM1 (ProSci Inc.), Rabbit anti-TRPC1 (Alomone Labs), Rabbit anti-TRPV6 (Santa Cruz Biotechnology) and Rabbit anti-β-actin (Santa Cruz Biotechnology) and horseradish peroxidase-conjugated secondary antibodies (Santa Cruz Biotechnology).

### Cell culture

LNCaP, LNCaP-C4.2 and PC-3 PCa cell lines, obtained from the American Type Culture Collection (ATCC, Manassas, VA, USA), were cultured in RPMI 1640 and serum as described by Gackière *et al*. (Gackiere et al. [2006]). For BPA experiments, the cells were treated with Phenol red-free RPMI 1640 containing charcoal-stripped Foetal Calf Serum (FCS) (CS-RPMI). In order to avoid the interference between BPA added for our studies and those leached from RPMI 1640 commercialized in polycarbonate bottles, the medium was prepared in glass bottles using RPMI 1640 powder commercialized by SIGMA (L’Isle d’Abeau, France) in ultrapure water and then filtered on 0.2 μm filters (Thermo Scientific Nalgene, Fontenay-sous-Bois France).

### RT–PCR analysis of mRNA expression

Total RNA isolation and RT-PCR experiments were performed as described earlier (Roudbaraki et al. [1999]). The PCR primers (Orai1: 5’-CTTCTTCGACCTCGTCCTCCT-3’ and 5’-CGTAAGGCCAAAGCATGGAA-3’; β-actin : 5’-CAGAGCAAGAGAGGCATCCT-3’ and 5’-GTTGAAGGTCTCAAACATGATC-3’ used in this study were designed on the basis of established GenBank sequences and synthesized by Invitrogen (Carlsbad, CA, USA). The amplified PCR products were of 406 and 212 bp respectively.

### siRNA transfections

For siRNA experiments, equal numbers of cells from the same culture were seeded, transfected overnight with 20 nM of control siRNA (targeting Luciferase mRNA) (Eurogentec, Belgium), or raised against Orai1 mRNA (siOrai1) (5^′^-UGAGCAACGUGCACAAUCU (dTdT)-3^′^), using Hyperfect transfection reagent (Qiagen Inc., Courtaboeuf, France) in CS-RPMI containing 10% SVF, according to the manufacturer's instructions. Medium was changed after 24 h and cells were incubated for a further 48h with or without BPA, before performing calcium imaging experiments. We previously showed the efficiency of the siOrai1 used in the present study, in down-regulating the expression of the Orai1 protein in LNCaP cells (Flourakis et al. [2010]).

### Orai1 immunofluorescence studies

The protein expression studies of the ion channels in PCa cells were determined by indirect immunofluorecence analysis performed on acetone-fixed cells. Cells grown on glass cloverslips were incubated with PBS containing 0.2% BSA, 0.1% TritonX-100 and 5% donkey serum, for 30 min at room temperature, in order to block the non-specific bindings and to permeabilize the cells. They were then incubated overnight at 4°C with PBS/5% non-immunized serum containing a 1:50e dilution of the primary affinity-purified rabbit anti-Orai1 polyclonal antibody. Cells were then washed with PBS and were incubated with the secondary Alexa fluor 488-labeled anti-rabbit IgG (A-21206; Molecular Probes; dilution 1:2000e) diluted in PBS for 1 h at room temperature. After rinsing three times in PBS, the slides were mounted with Mowiol and the distribution of the labelled proteins was analysed by confocal immunofluoresccence microscopy (Zeiss LSM 510; acquisition parameters: objective 40x/1.3; thickness of confocal slide, 1 μm).

For the immunofluorescence studies of Orai1 protein in human PCa, tissues were obtained from consenting patients following local ethical considerations. The tissues were diagnosed as cancerous or not by anatomopathological examinations. Tissues were from patients prior to any anticancer therapy (chemotherapy, radiotherapy) and were obtained following an office procedure, frozen in liquid nitrogen-cooled isopentane and kept in “Tissue-Tek®” at −80°C before 10 μm cryosections were carried out at −20°C with a cryostat and mounted on glass slides for immunofluorescence studies. All experiments involving patient tissues were carried out under approval number “CP 01/33”, issued by the “Comité Consultatif de Protection des Personnes dans la Recherche. The immunofluorescence experiments for the detection of Orai1 on 7 μm cryosections was carried out following the same procedure as for the PCa cell lines, using anti-Orai 1 antibody and analysed by confocal microscopy.

### Western blot assay

Cells cultured at 80% confluence were harvested and total proteins extracted. 40 micrograms of each sample were analysed by SDS-PAGE on 10% acrylamide and processed for western-blotting using antibody as described by (Vanoverberghe et al. [2004]) using BKca (Alomone, 1:500e), TRPV6 (Alomone, 1:500e), TRPC1 (Alomone, 1:500e), Orai1 (ProSci 1:500e), STIM1 (ProSci, 1:500e), IKCa1 (Santa Cruz, 1:200e). Western blotting was performed with an ECL chemiluminescence kit (Millipore). Quantitative evaluation of protein expression was performed using ImageJ software.

### [Ca^2+^]_i_ measurements

Cells were grown on glass coverslips for [Ca^2+^]_i_ imaging experiments and, before each experiment, the cells were loaded with Fura-2, by adding 2 μM Fura-2 AM (Fura-2 Acetoxymethyl esther) (Calbiochem, Meudon, France) to the culture medium for 45 min at 37°C. The cells cells were then washed three times in HBSS (Hanks Balanced Salt Solution; 142 mM NaCl, 5.6 mM KCl, 1 mM MgCl_2_, 2 mM CaCl_2_, 340 μM Na_2_PO_4_, 440 μM KH_2_PO_4_, 10 mM Hepes, 5.6 mM glucose and buffered to pH 7.4). When a Ca^2+^-free medium was required, CaCl_2_ was omitted and replaced by equimolar MgCl_2_. The fluorescent intensity of Fura-2 in each cell was monitored and recorded at 340 and 380 nm. To represent the variation in the intracellular free calcium concentration, either the fluorescence intensity ratio represented by F340/F380 was used as an indicator of changes in cytosolic Ca^2+^ concentrations, or a calibration was used to represent such variations in nM. All measurements shown are averages of 35–45 cells from a minimum of four experiments on different cell cultures.

### Cell migration assays

Cell migration assays were performed in duplicate in modified Boyden chambers. These assays consisted in counting cells migrating through a porous membrane with 8 μm pores (BD Biosciences, Oxford Science Park, Oxford, UK). After trypsinisation, cells in suspension (1 × 10^5^) were loaded into the upper chamber in phenol-red RPMI without FCS. The lower chamber contained RPMI and 10% charcoal-stripped (CS)-FCS (CS-RPMI). The upper and lower chambers contained the same concentration of BPA when tested. After 16 h to 24 h at 37°C in a 5% CO_2_ incubator, cells that had attached but not migrated were scraped from the upper surface, the membranes were fixed in 70% methanol at −20°C and the migrated cells were stained for nuclei with Hoechst 33342 dye (1 μg/mL) (blue fluorescent) and evaluated by counting cell nuclei in 10 randomly chosen fields under fluorescence microscopy. The results are presented as a percentage of control (CTL), where cells were incubated in CS-RPMI culture medium alone. Alternatively, the Wound Healing Assay was also used to study PCa cell migration. The cells were seeded in a 12-well plate (15 × 10^4^). After the cells formed a confluent mono-layer, scratches were performed using a 100 μl tip. The wells were washed with PBS followed by the addition of BPA at different concentrations in CS-RPMI. The closure of scratch was analyzed under the microscope and images were captured 1 and 15 or 24 h after incubation in the presence or absence of BPA.

### Statistical analysis

Plots were produced using Origin 5.0 (Microcal Software, Inc., Northampton, MA). Results are expressed as mean ± S.E. Statistical analysis was performed using unpaired *t* tests or ANOVA tests followed by either Dunnett (for multiple control *versus* test comparisons), or Student-Newman-Keuls post-tests (for multiple comparisons). The Student’s *t*-test was used for statistical comparison of the differences and p < 0.05 was considered significant.
